# A scalable diffraction-based scanning 3D colour video display as demonstrated by using tiled gratings and a vertical diffuser

**DOI:** 10.1038/srep44656

**Published:** 2017-03-17

**Authors:** Jia Jia, Jhensi Chen, Jun Yao, Daping Chu

**Affiliations:** 1 Centre for Photonic Devices and Sensors, Department of Engineering, University of Cambridge, 9 JJ Thomson Avenue, Cambridge CB3 0FA, U. K; 2 Huawei Technologies Co. Ltd, Huawei Industrial Base, Bantian Longgang, Shenzhen, Guangdong 518129, P. R. China

## Abstract

A high quality 3D display requires a high amount of optical information throughput, which needs an appropriate mechanism to distribute information in space uniformly and efficiently. This study proposes a front-viewing system which is capable of managing the required amount of information efficiently from a high bandwidth source and projecting 3D images with a decent size and a large viewing angle at video rate in full colour. It employs variable gratings to support a high bandwidth distribution. This concept is scalable and the system can be made compact in size. A horizontal parallax only (HPO) proof-of-concept system is demonstrated by projecting holographic images from a digital micro mirror device (DMD) through rotational tiled gratings before they are realised on a vertical diffuser for front-viewing.

A 3D images display supporting all depth cues can generate directional voxels (volume pixels), which emits light with different colours and variable brightness in different directions. This information of spatial images can be described as the wave front in holographic displays, or as the five dimensional vector field in light field displays^1^.

Digital holographic displays, which use computer generated holograms to implement 3D image displays, rely on spatial light modulators (SLMs), which support phase modulation to form the directional light^2^. The implementation of holographic displays is limited by SLM technology on many aspects. One is the limited angle of light distribution due to the pitch size of SLM pixels. Another is the limited space-product bandwidth (pixel count per frame), so only limited amount of information is delivered during a given period of time. This can be improved gradually along with SLM technology development. However, at present physically tiling multiple SLMs^3^,^4^,^5^,^6^, using a high speed SLM^7^, or combining both approaches^8^ is necessary to support the required bandwidth. At the same time, to practically implement holographic displays, a scanning/tiling mechanism is often employed to distribute such amount of information.

On the other hand, light field displays using incoherent light have an advantage of being free from the speckle noise in holographic displays^9^,^10^,^11^,^12^ while maintaining the potential to generate full 3D depth cues^13^,^14^. Similarly, for light field displays to distribute the information needed and produce directional light effect, a light distribution system (scanning/tiling mechanism) is required. Such a system can be a micro-lens array^15^, a rotation mirror^16^,^17^, an rotation off-axis lens^7^,^18^, or simply a combination of multiple projectors^4^,^19^,^20^.

Attempts have been made to distribute the image information in space uniformly and efficiently using optical means. QinetiQ used micro-lens array to replicate image information optically, but it suffered from high energy loss^21^. In integral imaging^22^ (another form of light field displays), micro-lens array is also used and the outcome suffers from the trade-off between the image resolution and viewing angle.

There are also mechanical methods to distribute image information, using such as galvanometers, polygon mirrors or rotating plates. Galvanometers can achieve temporal tiling^8^,^23^ of the information from a digital micro mirror device (DMD) or a liquid crystal on silicon (LCOS) device, but the image size and the overall frame rate are limited by the rotation mechanism. To support a large image size, a large mirror is necessary but it will increase the weight of rotation screen, slow down the scanning speed, make it difficult to distribute the high amount of information bandwidth of a DMD, resulting in overlapping and the waste of information^24^,^25^,^26^. Another problem of using a galvanometer is the fly-back issue, as the scanning is always forward and backward rather than uni-directional. It causes uneven frame rate for different viewing positions if only one line is scanned^27^.

There are other systems using a polygon mirror to manage the scanning^28^. One advantage of using a cylinder prism/polygon mirror is that there is no flay-back issue. However, it suffers from the weight issue. For a 100 mm × 100 mm size side mirror on an 8 facets prism, the total size of the prism would be larger than 300 mm × 300 mm × 100 mm. Driving it rapidly as required by video projection will need a powerful motor, which will make the system bulky, heavy and noisy.

Holographic optical element^29^ or off-axis Fresnel lens^7^,^18^,^30^ was also used to deliver images toward different directions through an unidirectional rotation. This structure can be made light-weight, easy to rotate (requiring a less powerful motor) and able to support the scanning for all the information amount on one DMD device. However, most of the systems based on this structure produce the circle viewing area which is not suitable for front-viewing systems. A recent work reported the use of a rotating off-axis lens and a vertical diffuser to distribute information from two DMDs for a front-viewing horizontal-parallel-only (HPO) holographic display^31^. It demonstrated only single colour images and it suffered from the fly-back issue.

In above, key limitations of the existing information distribution mechanisms were discussed. Here we propose the use of diffraction features in light field displays for image information distribution and management of directional lights. In principle, these features can be devices or systems which support changeable gratings, such as SLMs. Future SLMs have the potential to be made in sub-micro pitch^32^ and to be driven in fast speed^33^, which will realise the compact information distributor. In this work, rotational tiled gratings, which is slim and light-weight, are used to show the feasibility and potential performance. Together with a vertical diffuser, a proof-of-concept HPO display is demonstrated to deliver a full colour front-view 3D video.

## Results

### Experiment setup

**Figure 1:**
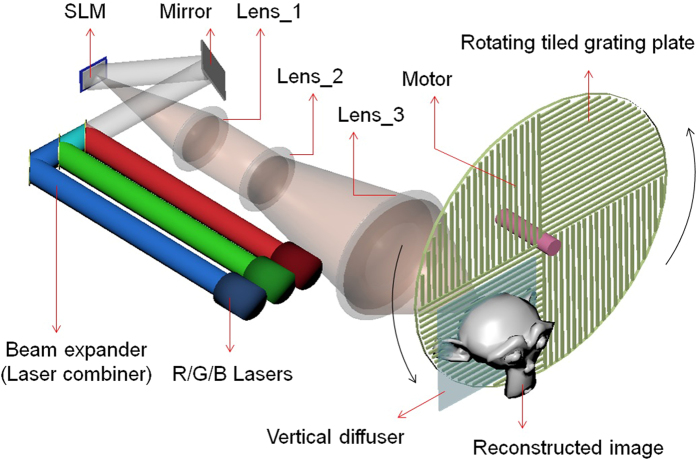
Illustration of the proposed system. The head shown here is adapted from the built-in 3D model of Blender3D^34^, an open-source graphics software.

The proposed display system comprises a high speed DMD, a motor driven rotation screen which is attached with tiled gratings, a vertical diffuser, lasers and lenses, as shown in Fig. 1. DMD is chosen as the information provider because it provides the highest bandwidth (both pixels per second and bits per second) among all existing available SLMs. Images projected from the DMD are distributed to different viewing angles by the rotation plate. 3D images with parallax can be formed once parallax images are properly projected to their corresponding viewing directions.

Based on the proposed system, a holographic stereogram 3D display system with a large viewing angle is developed. The DMD in use (V-9501 VIS) has a HD resolution of 1920 × 1080, a pixel pitch of 10.8 μm, and a written maximum frame rate of 17 kHz. This specification can deliver 35.2 Gpixel/second at most. However, the fastest frame period in our test is 75 μs, equivalent to 13.5kHz, and this may be related to the device controlling board model. It makes the available information amount at 28.0 Gpixel/second. In operation, the amplitude Fourier holograms are calculated by a view-dependent layer-based method^35^ and then converted to binary holograms. The DMD is uploaded with these calculated binary holograms to project different views in a time-sequential manner. By using holograms instead of binary images, DMD’s binary frames can deliver grey level images at the cost of degrading image quality and introducing speckle noise.

The red, green and blue lasers in use illuminate the DMD sequentially, and their wavelengths are 660 nm, 520 nm and 450 nm respectively. Lenses as labelled in Fig. 1 are used to reconstruct (Lens 1) the Fourier holographic images and magnify (Lens 2 & 3) images coming from the DMD, with Lens 1: a focal length of 500 mm and aperture of 2 inches in diameter, Lens 2: a focal length of 125 mm and aperture of 2 inches in diameter, and Lens 3: a focal length of 400 mm and aperture of 200 mm in diameter. A filter is put at the focus point of Lens 2 to block high orders.

The rotation screen with the size of 300 mm × 300 mm is divided into 4 parts (N = 4). Each part stands for 150 mm × 150 mm and is attached with the grating of 1,000 lines per mm. Each grating is placed perpendicular to its neighbouring gratings. N (N ≥ 2) can be chosen flexibly for different applications. In our system, we choose N = 4 to prove the proposed method. The servo motor (SMH80S), which is provided by Kinco with a maximum speed of 3,000 rounds per minute (rpm) and an encoder resolution of 10,000/round (equal to 0.036 degrees/step), is used to drive the rotation screen. The rotation speed is 1,125 rpm for the frame rate of 75 Hz (when N = 4 is used).

The vertical diffuser, which has the specification of 0.2 × 40° diffusive angle, is put after the grating. All three main devices, including lasers, the DMD and the motor, are synchronized through the computer with the multi-functional data acquisition board (NI-USB-6341).

### Reconstructed 3D images and performance

A 3D model is designed for displaying. Holograms of different parallax images of this 3D model are calculated and then loaded on the DMD. Both DMD and lasers are synchronized with the motor to deliver parallax images into their corresponding viewing angles.

**Figure 2:**
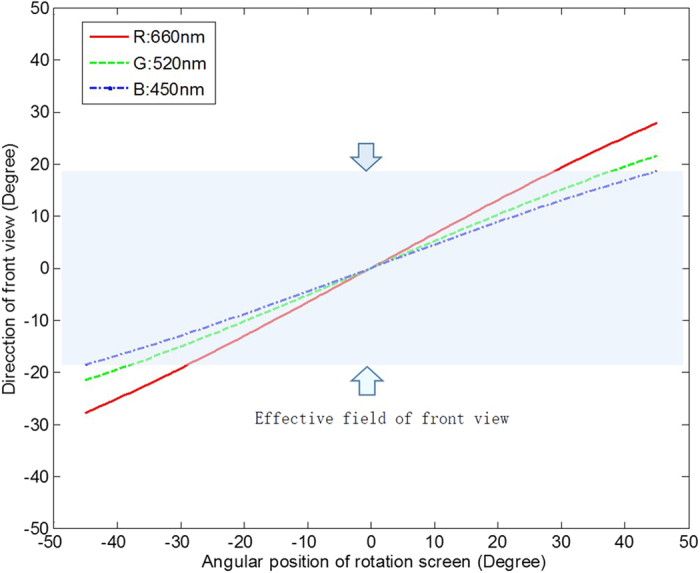
Relationship between rotation angle and viewing angle.

The relationship between the grating angular position β and the projected front-viewing direction θ for three wavelengths (R/G/B) are shown in Fig. 2. β ranges between −45° and + 45° in the system while we set 0° as the direction perpendicular to the grating plate. The front-viewing direction θ has total ranges of ± 27.5°/ ± 21.5°/ ± 18.5° for R/G/B respectively. In order to support R/G/B simultaneously, the viewing angle is limited by the blue channel, and the effective viewing angle is ± 18.5°, as shown in the shallow coloured band in Fig. 2. (Details about their corresponding equations are provided in the method section).

**Figure 3:**
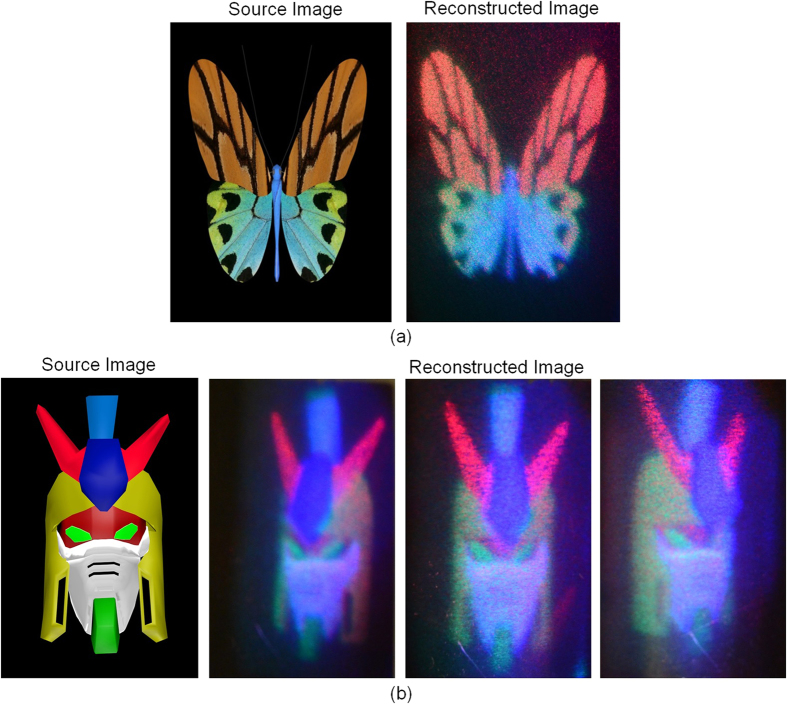
(**a**) The source image and the reconstructed image of a butterfly with the size of 60mm × 30 mm, and (**b**) the source image and three views of a reconstructed robot head from −18.5° to + 18.5°. [The robot head shown is adapted from the 3D model provided by Fabelar (user name) from CGTrader^36^. The butterfly shown is our modification of the 3D model from 3D66^37^, a 3D model open-resource website.].

On the other hand, 60 views are sampled for the whole scanning range for all R/G/B images. The horizontal viewing angle is ± 18.5°, in which R/G/B colours are supported. The vertical viewing angle is more than 40°, decided by the practical common diffusive angle from the diffuser (details are provided in the discussion section). The DMD runs at 13.5kHz and provides the overall 3D image frame rate at 75 Hz (13,500 = 3 (R/G/B) × 60 (views) × 75). The final image size is 60 mm × 30 mm. In sum, the system effectively distributes information amount of 11.6 Gpixels/second (see the Methods section for detailed calculations) from a DMD to a large viewing angle and provides holographic 3D images of 60 mm × 30 mm ×  ± 18.5° (HPO) with 3 colours at 75 Hz, as shown in Fig. 3. The results show that the horizontal parallax 3D images can be observed in a large viewing area and the motion parallax is continuous. Video results are also provided in the Supplementary Part. The reasons which cause colour mismatch are discussed in the Supplementary. Note that the sizes/angles are directly measured in space, the frame rate is measured by projecting on/off images and the frequency is detected by a photo sensor.

## Discussion and Future work

The main advantage of the proposed approach compared to the existing holographic displays comes from the use of tiled gratings and front-viewing projection. They allow information to be distributed horizontally without suffering from fly-back issue and reduce the load of the motor. As a result, the bandwidth of a rapidly-driven SLM, DMD, can be used fully for visual experience (in terms of image size, viewing angle, colours and frame rate).

The proposed approach is scalable. Although the ratio between the projected image and the rotation screen size is not very large, which is the natural limitation of this approach, the total image size can be enlarged by using a larger rotating screen. Currently, we haven’t driven our motor to its limit, and we can even switch to a more powerful motor to drive a larger rotating screen at the target speed. In this way, the information distribution capability will be significantly enhanced to more than that a DMD can support, and multiple DMDs can be used in future development. In addition, the approach proposed here also has the flexibility to trade between the overall frame rate and the viewing angle by adjusting the number of tiled grating.

Issues related to the image quality need to be addressed. The result images show limited sharpness and image quality. There are multiple reasons involved, mainly including 1) the accumulated synchronization error, which is caused by the clock mismatch between the DMD and the motor driver control; 2) the speckle noise, which is caused by the use of holograms reconstructed from laser sources; 3) the binary holograms in use, as the use of a DMD device for its high frame rates is at the cost of image quality degradation due to its binary nature; 4) the use of continuous lasers, which causes a certain degree of image dragging and crosstalk; 5) the use of a vertical diffuser; 6) the unevenness of the grating and the polycarbonate board, and 7) the zero-order interference, which is caused by the low diffraction efficiency of the grating. All these factors are practical issues in implementation, and detailed discussions are provided in the Supplementary Part.

In addition to the ultimate goal of developing a rapidly tuneable grating on SLM to realize a compact information distribution system physically, other further improvements may include upgrading components and scaling up the system to obtain the 3D images with higher quality, larger size and viewing angle. The DMD used in above is driven at 13.5 kHz, and it can be increased to 17 kHz by updating the controlling board according to the manufacturer’s latest module specification. At the same time, it will be desirable to use three DMDs to avoid the information loss for colour angle matching. By using three sets of magnification optics on DMDs respectively, each channel can match in size and projection angle without having blank areas. This will not only further enhance the information usage efficiency, but also further expand the effective information bandwidth. It can potentially transmit information more than 50 Gpixel/second. Furthermore, the driving motor hasn’t reached its limit yet since the plate is pretty light-weight in this structure. Therefore, the system can afford a larger rotational plate to project a large size image with the same viewing angle and frame rate, which can also be used on multiplexing imaging systems and star-trackers^38^,^39^,^40^. Finally the image quality can be improved by using vibrating diffusers^41^,^42^ to reduce the visible grainy-like noise. Moreover, switchable LC devices may be used in the future as electrically controlled diffusers in place of the mechanical vibrating ones, as long as they can respond fast enough.

## Methods

### Tiled multiple gratings in rotation screen and designing of front viewing

The rotation plate is divided into N parts (N ≥ 2), each of which is attached with one grating (N = 4 is shown as an example in Fig. 1). Each grating represents an angle of 360/N degrees. Assuming images are coming from a fixed direction and location while the grating plate keeps rotating, the light will be directed into different angles. On the rotation plate, once light passes the boundary of one grating and reaches to the neighbouring grating during the plate rotation, the diffracted light will immediately return back to the start of the scanning route.

**Figure 4:**
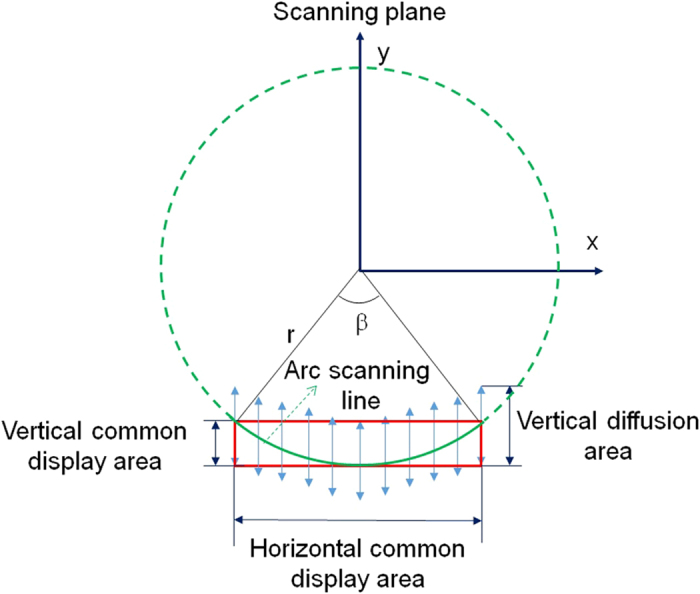
Illustration of the effective front-viewing zone. The green line means the arc scanning route without using a vertical diffuser.

The scanning route, which is the light track on the grating plate, is not a straight line but an arc during plate rotating. It means the diffraction light scanning route is not suitable for front-viewing display. Therefore, a vertical diffuser, which mainly diffuses light into vertical direction, is put right after the grating to extend the vertical viewing zone, so that the common part of the overall diffusive area is suitable for front-viewing display. The viewing zone illustration is shown in Fig. 4.

Comparing with the circle-viewing scanning and other front-viewing scanning systems, there are several advantages in the proposed method, including: (1) Low powerful motor requirement. The use of multiple gratings can reduce the necessary rotation screen speed down to 1/N times for the same target frame per second (fps) because all the gratings represent the same scanning line. Therefore, it is easier to drive a large rotation plate for the large size image. (2) Flexible viewing range. N can be any number (≥2) deciding the viewing ranges, depending on the applications. (3) High information distribution efficiency. The scanning is unidirectional and has no fly-back issue.

### Horizontal front-viewing angle

The use of the tiled gratings as the information distributor comes with some unique properties on the horizontal front-viewing angle as discussed following.

**Figure 5:**
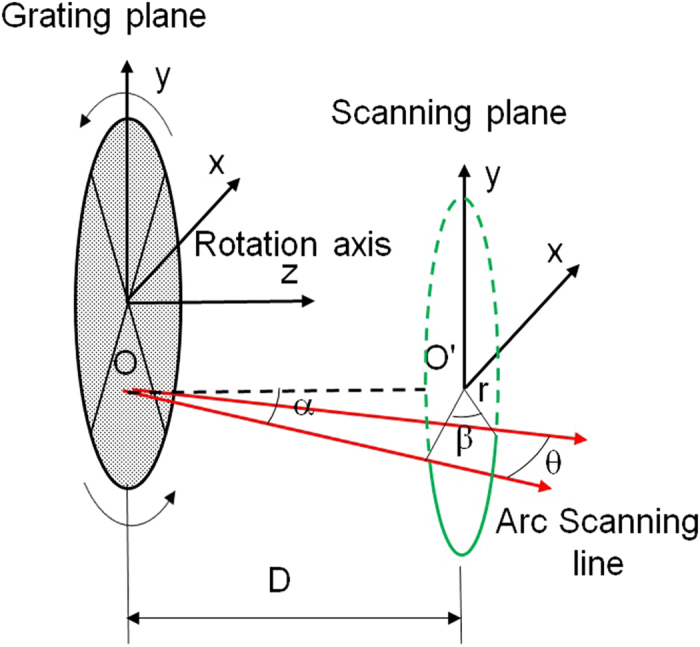
The horizontal viewing zone.

With the tiled gratings on the rotation plate, the overall horizontal viewing angle is determined by the diffraction angle of grating and gratings number N which decides the rotation angle. Fig. 5 illustrates the relation between diffraction angle, rotation angle and viewing angle.

O is the diffraction point on the grating. D is the distance between the grating and the diffuser. *r* is the radius of scanning line. The front-viewing angle θ can be calculated by the follow equation:


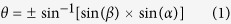


where β is the angle position which ranges from ± 180/N degrees (N ≥ 2) and β = 0 when grating lines parallel to horizontal direction. α is the diffraction angle of the grating, which is wavelength dependent. The maximum front-viewing angle is 2 × α according to Equation (1), when the rotation angle is ± 90° (equal to N = 2). Note that gratings with a higher resolution (larger diffraction angle) can support a larger front-viewing angle.

The relationship between the viewing angular pitch and the rotation angular step is shown in Equation (2) which obtained from the differential of Equation (1).


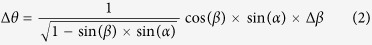


where Δθ is the angular pitch, which is defined as the separation angular distance between two neighbouring views; Δβ is the rotation angular step which is related to the rotation speed of motor. It shows that the viewing angular pitch Δθ is not linear to Δβ, neither linear to horizontal viewing direction. This phenomenon leads to the analysis in the next sub-section.

### Angular pitch criteria

**Figure 6:**
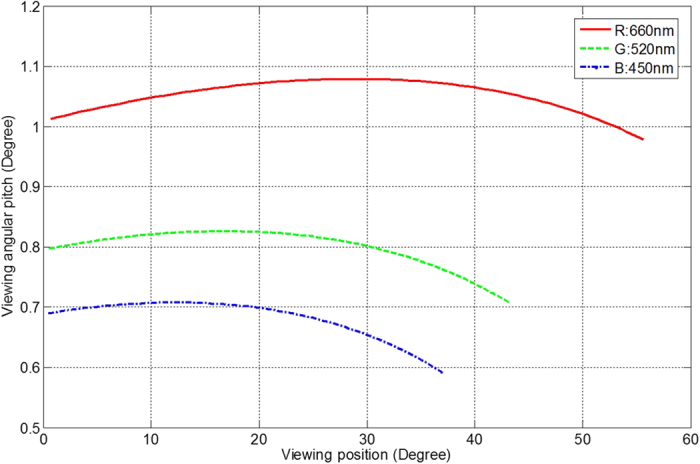
The relationship between the angular pitch and the viewing position.

According to Equations (1) and (2), the change of viewing angle along the rotation is not constant. It means that the angular resolution is at its worst when the change of viewing angles is at the maximum. The angular pitch is defined as the change of viewing angle for one view to another. Since the number of views for each channel is fixed, and the views are evenly distributed along the rotation angle (β), the corresponding front-viewing angle can be found out for each view, as shown in Fig. 6, in which Δβ is 1.53 ° = 90 ° /(60-1 views). The maximum viewing angular pitch (worst angular resolution) is 1.079° at the peak of red channel, which defines the angular resolution and limits the image depth.

### Vertical viewing angle

Each colour channel of 3D images has their own square region for front-viewing as shown in Fig. 4 which only shows one channel. Each channel’s square front-viewing region is shifted with a degree to each other due to the diffraction for different wavelengths. The vertical diffuser with 40° vertical diffusing direction is used to provide a large enough common area to support R/G/B simultaneously. Since the diffraction angle difference between blue (450 nm) and red (660 nm) light is less than 10° after the grating in use (1000 lines/mm), the common area is larger than 30°. In practice, the vertical diffuser in use spreads the light vertically in an angular range around 80° which is much larger than 40° as in the spec, so the common area becomes large to around 70°. This significantly large vertical diffusion brings up the zero-order interference for the viewing at the centre (discussions in Supplementary).

### Colour angle match and its impact on the effective information amount

To achieve colour angle match, three colours should cover the same viewing angle within the scanning period. In the vertical axis, vertical diffuser extends the viewing angle widely for red, green and blue, so that the common area is large enough to cover each other. In the horizontal axis, the viewing angle is determined by both the rotation angle and the diffraction angle which is related to wavelength. This means, images with different colours under the same rotation angle will be delivered to different directions. Fortunately, the horizontal scanning range is large for red, green and blue channel, and there is a big common range we can use. Images with different colours can be projected to the same direction if the grating rotates to their corresponding angle. Therefore each hologram should consider its corresponding channel and the corresponding horizontal projected direction (related to the motor and the grating rotation) to apply the correct viewing angle on the graphics rendering in the calculation. The necessary rotation angle of different wavelengths, β_λ_, can be derived from Equation (1) and shown in Equation (3), in which α_λ_ means the diffraction angle for different wavelengths.


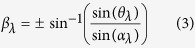


In our system, the physical range width of rotation angle is 90°. This means we set the range of β_450_ to be −45°~ +45° with which the blue channel produces a viewing range of −18.5° ~ +18.5°, equal to 37° viewing angle, according to equation (1). To match 37° viewing angle, red and green channels only need to rotate 57° (range width of β_660_) and 76°(range width of β_520_) respectively, which means no image should be projected outside these range (blue channel doesn’t support these ranges). Effectively, some frames will be blank and the hardware information amount is not used.

For blue channel, all 60 views are assigned values and delivered to 37° viewing angle (90° rotation scanning). For green channel, 51 views out from 60 views are assigned values while other 9 edge views are left empty, 37° viewing angle (76° rotation scanning). For red channel, 38 views out of 60 views are assigned values while other 22 edge views are left empty, so that those views with values make up 37° viewing angle (57.4° rotation scanning). Therefore, the effective information becomes (60 + 51 + 38)× 75 × 1920 × 1080 × 0.5 (binary) = 11.6 Gpixels/second.

It is noted that the numbers of views for different colours are different at the common viewing zone. Ideally, the number of views should match each other to obtain the correct colour angle match, which means minimum views among three channels should be set as the common views number. In our experiment, to match the 60 views of blue channel, the views for red and green channel outside of 38/51 views should be empty. This means the frames usage of DMD is reduced. The parallax differences between adjacent parallax images are too small to be identified visually. Therefore, all 60/51/38 views for blue/green/red channel are assigned values, and the colour matches to each other direction-to-direction.

It is also noted that R/G/B channels are directed into different directions after the grating and there is a small distance between the grating and vertical diffuser, hence there is a slight position mismatch, which is not a constant and it varies according to the wavelength and the viewing direction. This mismatch is measured and compensated in our hologram calculation.

## Additional Information

**How to cite this article:** Jia, J. *et al*. A scalable diffraction-based scanning 3D colour video display as demonstrated by using tiled gratings and a vertical diffuser. *Sci. Rep.* **7**, 44656; doi: 10.1038/srep44656 (2017).

**Publisher's note:** Springer Nature remains neutral with regard to jurisdictional claims in published maps and institutional affiliations.

## Supplementary Material

Supplementary Information

Supplementary Video1

Supplementary Video2
