# Corrigendum: A scalable diffraction-based scanning 3D colour video display as demonstrated by using tiled gratings and a vertical diffuser

**DOI:** 10.1038/srep46648

**Published:** 2017-05-03

**Authors:** Jia Jia, Jhensi Chen, Jun Yao, Daping Chu

Scientific Reports 7: Article number: 4465610.1038/srep44656; published online: 03 17 2017; updated: 05 03 2017

The original version of this Article contained a typographical error in Reference 6, which was incorrectly given as: Yaracs, F., Kang, H. & Onural, L. Circular holographic video display system*. Opt. Express* **19**, 9147–9156 (2011). This has been corrected in the HTML and PDF versions of this Article.

