# Supplementary material for: A scalable diffraction-based scanning 3D colour video display as demonstrated by using tiled gratings and a vertical diffuser
Source: Sci Rep. 2017 Mar 17;7:44656. doi: 10.1038/srep44656 (PMC5356339; doi:10.1038/srep44656)
Supplement: Supplementary Information [file srep44656-s1.doc]

**Supplementary to “A scalable diffraction-based scanning 3D colour video display as demonstrated by using tiled gratings and a vertical diffuser”**

**Jia Jia^1^, Jhensi Chen^1^ (equal contributor), Jun Yao^2^, Daping Chu^1^,***

*^1^Centre for Photonic Devices and Sensors, Department of Engineering, University of Cambridge,
9 JJ Thomson Avenue, Cambridge CB3 0FA, U. K.*

*^2^Huawei Technologies Co. Ltd, Huawei Industrial Base, Bantian Longgang, Shenzhen, Guangdong 518129, P. R. China*

**corresponding author: dpc31@cam.ac.uk*

**Colour intensity issue.**

The power levels of the lasers we used are different (100 mw for red, and 50 mw for green/blue), and their actual output powers are also slightly different to their specifications. Moreover, the diffraction efficiency of the DMD and gratings are also different for different wavelengths. Therefore, the laser powers are calibrated to deliver a better colour range. Fig.S1 compare between the colour before and after some initial adjustments. The results show the colour match is better after the calibration, though not ideal. This is because the grating in use is slightly distorted and its diffraction efficiency is not uniform, which causes various colour intensity mismatches across the whole viewing range. For further improvement, the three lasers with the same powers and the better grating with uniform diffraction efficiency for different parts should be used.


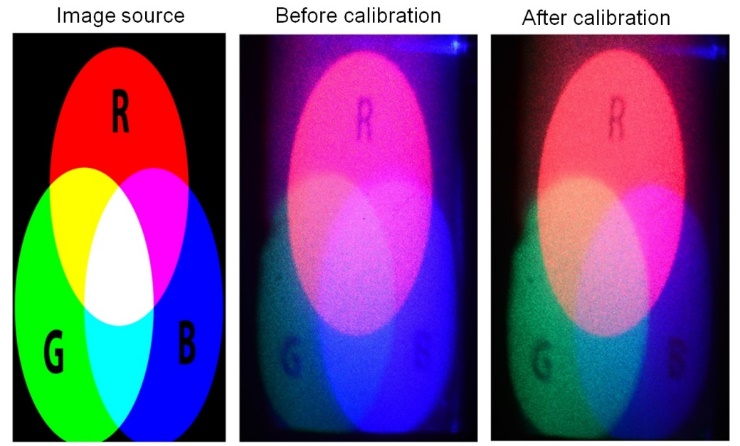


Figure S1 Laser power adjustment for better colour match.

**Discussion on the image quality.**

As mentioned in the paper, we will discuss 7 main factors which have the impact on image quality.

(1) There exists a synchronization error between the DMD and the motor. The DMD is running at its on-board clock while the motor is controlled by another digital- to-analogue control board (DtoA board) with its own on-board clock. Both the DMD and the motor are triggered by the DtoA board, but the DMD’s clock is different to the DtoA board’s clock, and the difference causes error which keeps accumulating. This error makes the reconstructed images not projected on the corresponding parts of grating and diffracted to the wrong viewing points. To reduce this mismatch error, we re-sychronize both DMD and the motor after a certain time of running. Re-synchronization makes the system stops for a short time, and causes a blink in projection, so it is not ideal to do it too frequently. To balance between the blink effect and the error accumulation we chose to re-sychronize at every 3-4 seconds. During which, the accumulated error unavoidably blurs the displayed image. This can be improved once the DMD control board is integrated with the main control board. As for the synchronization between the DMD and the laser, this is not an issue. The laser is triggered by the triggering signal produced by the DMD every time when it displays one frame. This triggering signal has a delay less than 2 μs, which causes no visible effect.

(2) The use of hologram reconstructed by lasers unavoidably introduces the speckle noise. There are many speckle noise reduction methods which can be applied in our system as our future work.

(3) The image quality is degraded due to the binary hologram. We choose to use the DMD as the SLM because its rapid rate of frames per second, but each frame is only a binary frame. This can only be improved when the grey level device is able to support the same rapid rate.

(4) The use of continuous lasers causes views dragging and crosstalk. Since lasers are continuous, each view content is dragging along the horizontal direction angularly for 1/3 of DMD frame duration (R/G/B are projected in sequence). This dragging makes the view content overlap to itself partially and to the adjacent view partially. This unavoidably reduces the image sharpness. Upgrading lasers to pulse lasers can reduce this effect.

(5) The vertical diffuser in use contributes to the dot-like grainy noise as seen in Fig.4. This may be improved by customizing the vertical diffuser.

(6) In our display system, 4 gratings are tiled and attached on the polycarbonate board to project images to different viewing points. Theoretically, the grating lines of 4 gratings should be the same, and their locations should be as the design. However in practice, grating lines are mismatched to each other due to number of reasons, including the assembling error, un-flatness of the polycarbonate board, attachment distortion (the holography diffraction films are slightly distorted when it is attached) and motor rotation wobbling (the polycarbonate board is not fully flat, and it causes board wobbling when the motor is driving at a high speed). In result, non-ideal scanning occurs and causes the images distortion and blurring. To calibrate errors mentioned above, the actually projecting directions and projected position are measured and compensated, but there is a limit we can push. We measured 50 points distortion across the whole scanning route statically and then compensate it back to the hologram calculation. However the actual projecting is running dynamically, and the measurement in static situation is not completely accurate. Besides, the number of 50 may not be fine enough as well. In addition, the wobbling is not a constant, but keeps changing. Therefore, the calibration mentioned above can only improve to a limit. To further improve the image quality, we should customize 1) the tiled gratings screen and 2) the coupler linking the grating plate and the motor shaft, and this is planned as the future work.

(7) The zero-order interference affects the quality of reconstructed images as well. Ideally, the zero-order light should be separated from the reconstructed images by using the vertical diffuser with the diffuse angle of 40 degree. However, the diffuser, although with specified as 0.2°×40°, actually diffuses light/images wider than 80°. Therefore, the zero-order is diffused with a large vertical angle, and it becomes visible at the horizontal centre view as recorded by the camera as a very bright light. For future improvement by customizing the vertical diffuser to limit the actual diffusive angle and using a grating of high diffraction efficiency, such as a blazed grating.

**Video results.**

We provide two video results as shown in Fig.S2. One is a static dice and the other is a moving robot head. In the video, one can notice that there is strong bright light when the camera moves to the central view, and this is caused by the zero-order interference, as mentioned in supplementary “Discussion on the image quality” section.


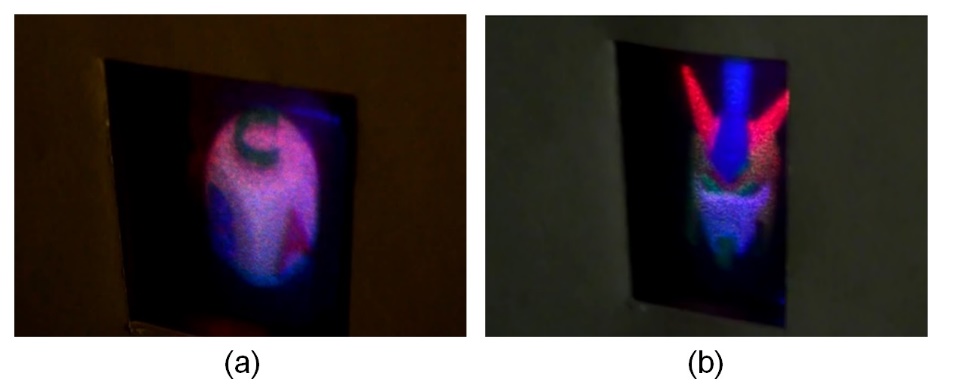


Figure S2 The videos for reconstructed images, (a) dice and (b) robot head. [The 3D model of the dice shown is provided by 3dregenerator (designer ID) from tf3dm^R1^, a 3D model open-resource website. The robot head shown is our modification of the 3D model provided by Fabelar (user name) from CGTrader^R2^.]

**Reference**

R1. Tfd3dm.com, Dice -3d model - .obj, <http://tf3dm.com/3d-model/dice-50306.html>, Date of access: 16 Jan 2017.

R2. CGTrader, Gundam free 3D Model MAX OBJ 3DS | CGTrader.com, https://www.cgtrader.com/free-3d-models/character-people/sci-fi/gundam, Date of access: 16 Jan 2017.
